# The effects of exercise and low-calorie diets compared with low-calorie diets alone on health: a protocol for systematic reviews and meta-analyses of controlled clinical trials

**DOI:** 10.1186/s13643-021-01669-7

**Published:** 2021-04-20

**Authors:** Sara Beigrezaei, Zeinab Yazdanpanah, Sepideh Soltani, Seyede Hamide Rajaie, Sahar Mohseni-Takalloo, Tayebeh Zohrabi, Mojtaba Kaviani, Scott C. Forbes, Julien S. Baker, Amin Salehi-Abargouei

**Affiliations:** 1 Nutrition and Food Security Research Center, Shahid Sadoughi University of Medical Sciences, Yazd, Iran; 2 Department of Nutrition, School of Public Health, Shahid Sadoughi University of Medical Sciences, Yazd, Iran; 3 Yazd Cardiovascular Research Center, Shahid Sadoughi University of Medical Sciences, Yazd, Iran; 4 School of Medicine, Bam University of Medical Sciences, Bam, Iran; 5 Faculty of Pure & Applied Science, School of Nutrition and Dietetics, Acadia University, Wolfville, NS Canada; 6 Department of Physical Education Studies, Faculty of Education, Brandon University, Brandon, MB Canada; 7 Centre for Health and Exercise Science Research, Head, Department of Sport, and Physical Education, Hong Kong Baptist University, Kowloon Tong, Hong Kong

**Keywords:** Diet, Diet plus exercise, Energy intake, Cardiometabolic markers, Mental health, Bone health, Systematic review, Meta-analysis

## Abstract

**Background:**

Exercise and weight loss diets are two independent non-pharmaceutical strategies used to improve several aspects of body composition and health. We plan to systematically review controlled clinical trials investigating weight loss diets alone compared to weight loss diets in conjunction with exercise on energy intake, body weight, body composition, cardiometabolic risk factors, sex hormones, and mental health.

**Methods and analysis:**

PubMed/MEDLINE, EMBASE, ISI (Web of Science), Scopus, and Google Scholar will be searched to retrieve potential controlled clinical trials investigating the effects of exercise in conjunction with weight loss diets compared with weight loss diets alone on energy intake, body weight and composition (fat mass, fat-free mass), anthropometrics (waist circumference), cardiometabolic markers, sex hormones [testosterone, estradiol, and sex hormone binding globulin (SHBG)], liver and kidney enzymes (alanine aminotransferase (ALT), aspartate aminotransferase (AST), gamma-glutamyl transferase (GGT), uric acid, blood urea nitrogen (BUN), glomerular filtration rate (GFR), quality of life, and depression in adults. The weighted mean difference (WMD) and its corresponding 95% confidence intervals (CIs) will be derived using random effects model. Several subgroup analyses based on follow-up duration, the health status of the participants, the diet used for weight loss, the exercise protocol, participants’ sex, and other possible variables will be conducted to explore possible sources of heterogeneity. Publication bias will be explored by inspecting funnel plots and by conducting asymmetry tests. Overall quality of the evidence will be assessed by using the Grading of Recommendations Assessment, Development, and Evaluation (GRADE) tool.

**Discussion:**

We envisage that this systematic review and meta-analysis will provide valuable information regarding the effectiveness of adding exercise to weight loss diets. No primary data is going to be collected; therefore, ethical approval is not required. The resulting manuscripts will be disseminated in peer-reviewed journals and at international and national conferences.

**Systematic review registration:**

The study protocol is registered in the International Prospective Register of Systematic Reviews (PROSPERO, Registration ID: CRD42020173434).

**Supplementary Information:**

The online version contains supplementary material available at 10.1186/s13643-021-01669-7.

## Background

The worldwide prevalence of obesity and associated metabolic abnormalities has resulted in a huge strain on health care systems [1, 2]. The increase in prevalence of obesity in recent decades is multifactorial; however, sedentary lifestyle and poor quality diets are proposed to be the two major contributing factors [2, 3]. Furthermore, obesity is associated with a reduced quality of life and higher risk factors for several diseases, such as metabolic syndrome, diabetes, cardiovascular disease (CVD), and cancers [4, 5].

Lifestyle modifications including changes in diet and physical activity are regarded as the main non-pharmacological and non-surgical strategies to treat obesity [6]. Modified dietary macro-nutrient intake leading to a hypocaloric diet is effective for weight loss over the short term and may be important for weight loss maintenance compared to exercise alone. Low-calorie diets not only reduce body weight but also improve cardiometabolic health, quality of life, and mental health [7–14]; however, it is proposed that weight loss diets might adversely affect bone health in adults [15]. It is also plausible that exercise may alter energy balance and influence body weight and health over time. Despite the well-known benefits of exercise, the increase in energy expenditure and the potential to decrease hunger and energy intake exercise alone does not seem to be effective at modifying weight status [16–18]. Beyond weight loss, exercise, may modulate metabolism and lead to an increase in muscle mass [18–21]. Furthermore, exercise (particularly weight-bearing exercise) may be effective at enhancing bone health [15].

In theory, exercise in conjunction with weight loss diets may be ideal to improve weight loss as well as appetite (i.e., energy intake), body composition, cardiovascular health and mental health [22–24]. However, controlled clinical trials have led to inconsistent results [25–29], with some studies demonstrating no additive effects of exercise [25, 26], while others found a greater effect with exercise for improving multiple cardiometabolic risk factors in obese adults [27, 28]. A number of clinical trials have revealed that subjects show a significant weight loss and reduction in energy intake during an exercise intervention, while others have shown less reduction in body weight due to an increase in energy intake [30–32]. These conflicting results were also observed on other health outcomes such as bone health, appetite, and mood [33–37].

A number of systematic reviews and meta-analyses have compared the effects of diet, exercise or both in specific health conditions [38, 39]. Hemmingsen et al. [38] reported no differences between the effects of diet alone or exercise alone compared to a standard treatment on the risk of type 2 diabetes mellitus and related complications. In addition, another systematic review evaluated the effects of diet or exercise or both on excessive weight gain during pregnancy and showed that diet or exercise alone and diet plus exercise during pregnancy appears to reduce the risk of excessive gestational weight gain [39]. Aside from the aforementioned reviews, we are not aware of any systematic review attempting to summarize the current evidence on other outcomes such as bone health, sex hormones, liver and kidney enzymes, quality of life, and depression.

## Objectives

In this study, we will describe the protocols used to systematically compare the effects of a low-calorie diet with a low-calorie diet plus exercise on energy intake, body weight and composition, anthropometric measures, cardiometabolic markers, bone health markers, sex hormones, liver and kidney enzymes, quality of life, and depression in adults.

## Methods

The present protocol is being reported in accordance with the reporting guidance provided in the Preferred Reporting Items for Systematic Reviews and Meta-Analyses Protocols (PRISMA-P) statement [40] (see checklist in Additional file 1). The study protocol is also registered in the International Prospective Register of Systematic Reviews (PROSPERO, Registration ID: CRD42020173434).

### Information sources and search strategy

The relevant articles will be identified in the following databases up to August 2020: PubMed/MEDLINE, EMBASE, ISI (Web of Science), Scopus, and Google Scholar using Medical Subject Heading (MeSH) and non-MeSH keywords. We will not apply any language or other restrictions. In addition, we will check the reference lists of all relevant studies to identify additional relevant articles. Unpublished studies will be identified by searching the websites indexing the preprints such as Research Square (https://www.researchsquare.com/) and the registered clinical trials approved by the World Health Organization (WHO). All abstracts of interest will be evaluated for further information by contacting the authors. The draft searches for the main databases are available in Additional file 2.

### Study selection

Two investigators will independently perform the study selection. All articles from electronic searches will be imported into the EndNote software (version: desktop, X7; Thompson Reuters, New York, USA) and duplicate studies will be deleted. Titles, abstracts, and full-text articles will be screened and cross-checked according to the eligibility criteria for study inclusion independently by 6 reviewers (Z.Y, S.S, S.B, SH.R, S.MT, and T.Z). Any disagreements will be resolved by discussion and consensus. The PRISMA flow chart will be presented to describe the process of the study selection.

Articles selected for full-text review must meet the following criteria:

(i) Participants must be a minimum 18 years of age and older and have a body mass index (BMI) ≥ 25 kg/m^2^ (pregnant and lactating women will be excluded);
(ii) Interventions must contain one arm in which participants receive an exercise intervention (i.e., aerobic or resistance) with a weight loss (i.e., hypocaloric) diet and one arm where participants only receive a weight loss diet (exactly according to the diet considered for the intervention group);
(iii) Interventions must be randomized or non-randomized controlled clinical trials with either a parallel, cross-over, or factorial design with at least 2 weeks of follow-up.

### Data extraction and management

The following data will be extracted using a predefined data extraction form by two independent investigators from the eligible studies and any discrepancy will be resolved by a third author:

#### Study and participant’s characteristics

The participants’ age, number of males and females, number of participants in the intervention and control group/period, the geographical location of the study, and the health condition of participants.

#### Intervention details

The study design (parallel/cross-over/factorial), number of study arms, the intervention duration, funding source(s), amount of calorie restriction, type of diets and exercise programs, intensity, frequency, compliance, and delivery of each exercise used for the intervention group.

#### Outcome measures

Data on baseline, post-intervention, or change from baseline mean ± standard deviation (SD) for energy intake, body weight, anthropometrics and body composition measures, blood glucose control markers (serum/plasma fasting glucose, insulin, insulin resistance markers including HOMA-IR and hemoglobin A1C), lipid profile [serum total cholesterol, triglycerides, low-density lipoprotein cholesterol (LDL-C), high-density lipoprotein cholesterol (HDL-C), and apoproteins], systolic and diastolic blood pressure, sexual hormones (testosterone and estradiol), SHBG, serum/plasma inflammation (hs-CRP, IL-6, and TNF-a), bone health markers, liver and kidney enzymes, depression, and quality of life, will be extracted for the intervention and control groups/periods. *P* values for within-group and between-group comparison will also be collected to calculate the change values.

### Assessment of risk of bias in individual studies

The eligible randomized trials will be assessed using the Cochrane collaboration’s risk of bias assessment tool considering seven domains: (i) random sequence generation (selection bias), (ii) allocation concealment (selection bias), (iii) blinding of participants and personnel (performance bias), (iv) blinding of outcome assessment (detection bias), (v) incomplete outcome data (attrition bias), (vi) selective reporting (reporting bias), and (vii) the dietary compliance as another possible source of bias in dietary interventions. Each study will be judged as low risk of bias, high risk of bias, or unclear risk of bias according to the mentioned domains [41]. The overall quality of studies will be classified as low risk (low risk for all domains), unclear risk (unclear for at least one domain), and high risk (high risk for at least one domain). As well, the non-randomized trials will be evaluated using risk of bias in non-randomized studies of interventions (ROBINS-I) tool [42]. According this tool, bias will be examined based on 7 domains (i.e., confounding factors, selection of participants, interventions classification, deviations from intended interventions, missing data, outcomes measurement, and selective reporting), and then reporting an overall risk of bias (i.e., low, moderate, serious, critical, or no information).

### Data analysis

The data for study characteristics, participants, outcomes, and findings will be used to build evidence tables for eligible studies to provide an overall description of included studies. The mean change values from baseline for the intervention (weight loss diet + exercise) and control group/period (weight loss diet alone) and their standard deviations (SDs) will be used to calculate the raw mean difference and standard error (SE) between the intervention and control. The hedges’ *g* (bias corrected standardized mean difference) statistic and corresponding SD will be calculated for outcome variables reported in different scales. The mean difference will be used as the effect size for meta-analysis. If the change values were not reported, we will calculate SD for the change values by selecting 0.5 as the reference correlation coefficient between baseline and end point values (*r* = 0.5) and to make sure that the meta-analysis was not sensitive to the selected correlation coefficient, all analyses will be repeated using 0.2 and 0.8 as the correlation coefficient. The weighted mean difference (WMD) and its corresponding 95% confidence intervals (CIs) will be derived using the random effects model which takes the between-study heterogeneity into account [43]. All statistical analyses will be performed using STATA, version 11.2 (Stata Corp, College Station, TX) and a two-sided *P* value less than 0.05 will be considered as statistically significant. If data cannot be meta-analyzed, we will summarize the articles and conclude on high-quality studies.

### Between study heterogeneity and subgroup analysis

The heterogeneity will be checked using Cochran’s *Q* test and *I*^2^ statistic (*I*^2^ is an estimate for between study variation to total meta-analysis variation ratio ranging from 0 to 100%) [44]. We will report Cochran *Q* test with a *P* value of < 0.05 considered statistically significant (heterogeneity). *I*^2^ with values of 0–25% and 75–100% will be taken to indicate low and considerable heterogeneity, respectively. To examine the potential sources of between-study heterogeneity, several subgroup analyses based on follow-up duration, the health status of the participants, the diet used for weight loss, the exercise, participants’ sex, and other possible variables will be conducted.

### Sensitivity analysis

The sensitivity analysis will be done by sequentially removing individual studies included in the meta-analyses to assess the robustness of the meta-analyses [45].

### Publication bias

In case there are fewer than 10 studies in a meta-analysis, we will construct a funnel plot to investigate the potential for publication bias for the primary outcome by visual inspection for asymmetry. If our meta-analysis involves 10 or more studies, publication bias will be evaluated by inspecting Begg’s funnel plots and Egger’s and Begg’s asymmetry tests [46]. Duval and Tweedie’s trim and fill analysis will be conducted if the publication bias becomes evident [47].

### Dealing with missing data

If data are missing, we will attempt to contact the authors through e-mails to obtain missing data or additional information twice, 1 week apart. The impact of missing data will also be evaluated in the sensitivity analysis. Additionally, we will describe the possible influences of missing data in the “Discussion” section of the resulting publications.

### Confidence in cumulative evidence

Overall quality of the evidence will be assessed by using the Grading of Recommendations Assessment, Development and Evaluation (GRADE) tool [48] with GRADEprofiler (GRADEpro) V.3.6 software, identifying the quality of evidence for each outcome as the extent to which one can be confident that an estimate of effect is near to the quantity of certain interest [46]. There are four levels used to rate the quality of evidence across trials in the GRADE system: very low, low, moderate, and high. Randomized clinical trials are categorized as high quality but can be downgraded due to limitation in study design, indirectness of evidence, imprecision of results, unexplained heterogeneity or inconsistency of results, or high probability of publication bias [48].

## Discussion

For decades, epidemiological and clinical studies have been elucidating the link between lifestyle modifications and health outcomes through different mechanisms [49]. Previous reviews have assessed the impacts of diet or exercise alone on energy intake and different health indicators, while there is no comprehensive investigation summarizing the evidence evaluating the effects of weight loss diets combined with exercise interventions on energy intake, anthropometric and body composition, blood glucose control, cardio-metabolic markers, and mental health. Nevertheless, this systematic review and meta-analysis might face several potential limitations. High heterogeneity between included studies might arise from differences in study characteristics not anticipated by authors or not explained at study level. The limited number of studies particularly RCTs with risk of bias on some outcome variables might lead to inconclusive results.

In this manuscript, we present the study protocol for a systematic review and meta-analysis to compare the effects of a low-calorie diet plus exercise with a low-calorie diet on risk factors associated with chronic diseases. Finally, this systematic review and meta-analyses will provide more information regarding the effectiveness of adding exercise to a weight loss diet.

## Supplementary Information

**Additional file 1.** PRISMA-P 2015 Checklist.

**Additional file 2.** Search strategies used to find related publications in PubMed, Scopus and ISI web of science.

## Data Availability

The studies included in the review will be available upon request.

## Abbreviations

ALT: Alanine aminotransferase
AST: Aspartate aminotransferase
BMI: Body mass index
BUN: Uric acid, blood urea nitrogen
CI: Confidence interval
CVD: Cardiovascular disease
GFR: Glomerular filtration rate
GGT: Gamma-glutamyl transferase
HDL-C: High-density lipoprotein cholesterol
hs-CRP: Highly sensitive C-reactive protein
IL-6: Interleukin 6
LDL-C: Low-density lipoprotein cholesterol
MeSH: Medical Subject Headings
PRISMA-P: Preferred Reporting Items for Systematic Reviews and Meta-Analyses Protocols
PROSPERO: International Prospective Register of Systematic Reviews
SD: Standard deviation
SE: Standard error
SHBG: Sex hormone-binding globulin
TNF-α: Tumor necrosis factor alpha
WHO: World Health Organization
WMD: Weighted mean difference
