# Supplementary material for: The effects of exercise and low-calorie diets compared with low-calorie diets alone on health: a protocol for systematic reviews and meta-analyses of controlled clinical trials
Source: Syst Rev. 2021 Apr 20;10:120. doi: 10.1186/s13643-021-01669-7 (PMC8059276; doi:10.1186/s13643-021-01669-7)
Supplement: Supplementary file 2 — Additional file 2. Search strategies used to find related publications in PubMed, Scopus and ISI web of science. [file 13643_2021_1669_MOESM2_ESM.docx]

### Additional file 2- Search strategies used to find related publications in PubMed, Scopus and ISI web of science.

| Database | Search strategy |
| --- | --- |
| PubMed | (Exercise[Mesh] OR Exercise[tiab] OR "resistance training"[Mesh] OR "resistance training"[tiab] OR "physical fitness"[Mesh] OR "physical fitness"[tiab] OR "Circuit-Based Exercise"[Mesh] OR "Circuit-Based Exercise"[tiab] OR "High-Intensity Interval Training"[Mesh] OR "High-Intensity Interval Training"[tiab] OR "Plyometric Exercise"[Mesh] OR "Plyometric Exercise"[tiab] OR hiking[tiab] OR "stationary bike"[tiab] OR "exercise therapy"[Mesh] OR "exercise therapy"[tiab] OR "tai ji"[Mesh] OR "tai ji"[tiab] OR "strengthening program"[tiab] OR Swimming[tiab] OR Walking[tiab] OR Yoga [Mesh] OR Yoga[tiab] OR "Tai Chi"[tiab] OR "weight-bearing"[Mesh] OR "weight-bearing"[TIAB] OR "weight lifting"[tiab] OR "weight lifting"[Mesh] OR Running[Mesh] OR Running[tiab] OR "Stair Climbing"[Mesh] OR "Stair Climbing"[tiab] OR Qigong[Mesh] OR Qigong[tiab] OR treadmill[tiab] OR "elliptical machine"[tiab] OR "free weights"[tiab] OR dumbbells[tiab] OR [gym](https://www.gymkituk.com/weights)[tiab] OR "static stretching"[tiab] OR "Muscle Stretching Exercises"[tiab] OR "Muscle Stretching Exercises"[Mesh] OR "dynamic stretching"[tiab] OR "Physical Education and Training"[Mesh] OR "Physical Education and Training"[tiab] OR "physical activity"[tiab] OR active*[tiab] OR activi*[tiab] OR "Physical Conditioning, Human"[Mesh] OR "Physical Conditioning"[tiab] OR Cycling[tiab] OR jog*[tiab] OR *jumping*[tiab] OR *aerobic*[tiab] OR training[tiab] OR "weight bearing"[TIAB] OR "muscle strength"[Mesh] OR "muscle strength"[tiab]) AND ("caloric restriction"[Mesh] OR "caloric restriction"[tiab] OR "weight reducing"[tiab] OR "dietary treatment"[tiab] OR "Reducing fat intake"[tiab] OR nutrition[tiab] OR "Weight Watchers"[tiab] OR diet[Mesh] OR diet*[tiab] OR hypocaloric[tiab] OR "Nutrition Therapy"[tiab] OR "Nutrition Therapy"[Mesh] OR calory[tiab] OR calori*[tiab] OR "Energy Intake"[Mesh] OR "Energy Intake"[tiab] OR "Weight Reduction Programs"[Mesh] OR "Weight Reduction Programs"[tiab] OR "weight management"[tiab] OR "weight reducing"[tiab] OR "weight control"[tiab]) AND (Randomized[tiab] OR random*[tiab] OR Intervention[tiab] OR "Clinical trial"[tiab] OR "Randomized controlled trial"[tiab] OR "Randomized controlled trials"[tiab] OR trial[tiab] OR Placebo*[tiab] OR "Double-blind"[tiab] OR "Single-blind"[Title/Abstract] OR "single blind"[tiab] OR "Random Allocation"[Mesh] OR "Random Allocation"[tiab] OR Randomised[tiab] OR "Randomised clinical trials"[tiab] OR "Randomised clinical trial"[tiab] OR controlled[tiab] OR "Clinical Trial"[Publication Type] OR "Clinical Trials as Topic"[Mesh] OR "Clinical Trials as Topic"[tiab] OR "Pragmatic Clinical Trial"[Publication Type] OR "Controlled Clinical Trial"[Publication Type]) NOT ("Cohort Study"[title] OR "Cohort Studies"[Mesh] OR "Cross-Sectional"[title] OR review[title] OR "Cross Sectional"[title] OR "in-vitro"[tiab] OR "in vitro"[tiab] OR "in-ovo"[tiab] OR cell[tiab] OR cells[tiab] OR “in vivo”[tiab] OR "in-vivo"[tiab] OR genes[Mesh] OR genes[tiab] OR gene[tiab] OR cistron*[tiab] OR genetic*[tiab] OR "Polymorphism, Genetic"[Mesh] OR "Genetic polymorphism"[tiab] OR "Polymorphism, Genetic"[tiab] OR "Mice"[Mesh] OR "Mice"[tiab] OR prevalence*[title] OR animal*[title] OR "birds"[MeSH] OR bird[tiab] OR birds[tiab] OR "fishes"[MeSH] OR "fishes"[tiab] OR mouse[tiab] OR rats[tiab] OR hamster[tiab] OR hamsters[tiab] OR pigs[tiab] OR pig*[tiab] OR swine[tiab] OR swines[tiab] OR piglets[tiab] OR piglet[tiab] OR "guinea pigs"[tiab] OR "guinea pig"[tiab] OR rabbits[tiab] OR rabbit[tiab] OR hares[tiab] OR hare[tiab] OR cats[tiab] OR cat[tiab] OR dogs[tiab] OR dog[tiab] OR canine[tiab] OR canines[tiab] OR canis[tiab] OR sheep[tiab] OR sheeps[tiab] OR monkey[tiab] OR monkeys[tiab] OR chimpanzee[tiab] OR chimpanzees[tiab] OR horse*[tiab] OR horses[tiab] OR pongo[tiab] OR chicken[tiab] OR chickens[tiab] OR reptile[tiab] OR reptilia[tiab] OR reptiles[tiab] OR Fish*[tiab] OR cow[tiab] OR cows[tiab] OR piglets[tiab] OR boar[tiab] OR boars[tiab] OR chickens[tiab] OR mare[tiab] OR mares[tiab] OR equine[tiab] OR "animal experimentation"[MeSH] OR "animal experimentation"[tiab] OR "Models, Animal"[Mesh] OR "Cohort Studies"[tiab] OR cohort*[title] OR Concurrent[tiab] OR incidence[title] OR "Cross-Sectional Studies"[Mesh] OR "Cross-Sectional Studies"[tiab] OR “Cross Sectional” [title] OR "invertebrates"[MeSH] OR mus[tiab] OR Rooster[tiab] OR Horses[mesh] OR goats[tiab] OR shrimp[tiab] OR sheep[tiab] OR crab[tiab] OR ducks[tiab] OR fowl[tiab] OR fowls[tiab] OR roots[tiab] OR bacteria[tiab] OR camel[tiab] OR Animal Model[tiab] OR laboratory animals[tiab] OR "Animals, Laboratory"[Mesh] OR "Qualitative Research"[Mesh] OR "Qualitative Research"[tiab] OR "Qualitative study"[tiab] OR “rat”[tiab] OR parakeets[tiab] OR parrot[tiab] OR parrots[tiab] OR donkey[tiab] OR donkeys[tiab] OR mule[tiab] OR mules[tiab] OR zebra[tiab] OR zebras[tiab] OR shrew[tiab] OR shrews[tiab] OR bison[tiab] OR bisons[tiab] OR buffalo[tiab] OR buffaloes[tiab] OR deer[tiab] OR deers[tiab] OR bear[tiab] OR bears[tiab] OR panda[tiab] OR pandas[tiab] OR "wild hog"[tiab] OR "wild boar"[tiab] OR ﬁtch[tiab] OR beaver[tiab] OR beavers[tiab] OR jerboa[tiab] OR jerboas[tiab] OR capybara[tiab] OR capybaras[tiab] OR child[MeSH] OR child[tiab] OR children[tiab]) |
| Scopus | ( ( ( ( ( ( TITLE-ABS-KEY ( ( randomi?ed W/7 trial* ) OR ( controlled W/3 trial* ) OR ( clinical W/2 trial* ) OR ( ( single OR doubl* OR tripl* OR treb* ) AND ( blind* OR mask* ) ) ) ) OR ( TITLE-ABS-KEY ( "Clinical trial" OR "clinical trial, phase i" OR "clinical trial, phase ii" OR "clinical trial, phase iii" OR "clinical trial, phase iv" OR "controlled clinical trial" OR "multicenter study" OR "pragmatic clinical trial" OR "randomized controlled trial" OR "random allocation" OR "clinical trials as topic" OR "4 arm" OR "four arm" OR "random allocation" OR randomized OR random* OR intervention OR trial OR placebo* OR randomised OR controlled ) ) ) ) AND ( ( ( TITLE-ABS-KEY ( ( nutrition W/2 "with" W/2 exercise ) OR ( nutrition W/2 plus W/2 exercise ) OR ( nutrition W/2 "in combination with" W/2 exercise ) OR ( nutrition W/2 "combined with" W/2 exercise ) OR ( nutrition W/2 "along with" W/2 exercise ) OR ( "nutrition and" W/2 exercise ) OR ( "nutrition+" W/2 exercise ) OR ( "nutrition +" W/2 exercise ) OR ( diet* W/2 "with" W/2 exercise ) OR ( diet* W/2 plus W/2 exercise ) OR ( diet* W/2 "in combination with" W/2 exercise ) OR ( diet* W/2 "combined with" W/2 exercise ) OR ( diet* W/2 "along with" W/2 exercise ) OR ( "diet and" W/2 exercise ) OR ( "diet +" W/2 exercise ) OR ( "diet+" W/2 exercise ) OR ( "nutrition therapy" W/2 "with" W/2 exercise ) OR ( "nutrition therapy" W/2 plus W/2 exercise ) OR ( "nutrition therapy" W/2 "in combination with" W/2 exercise ) OR ( "nutrition therapy" W/2 "combined with" W/2 exercise ) OR ( "nutrition therapy" W/2 "along with" W/2 exercise ) OR ( "nutrition therapy and" W/2 exercise ) OR ( "nutrition therapy +" W/2 exercise ) ) ) OR ( TITLE-ABS-KEY ( ( nutrition W/2 "with" W/2 "physical activity" ) OR ( nutrition W/2 plus W/2 "physical activity" ) OR ( nutrition W/2 "in combination with" W/2 "physical activity" ) OR ( nutrition W/2 "combined with" W/2 "physical activity" ) OR ( nutrition W/2 "along with" W/2 "physical activity" ) OR ( "nutrition and" W/2 "physical activity" ) OR ( "nutrition+" W/2 "physical activity" ) OR ( "nutrition +" W/2 "physical activity" ) OR ( diet* W/2 "with" W/2 "physical activity" ) OR ( diet* W/2 plus W/2 "physical activity" ) OR ( diet* W/2 "in combination with" W/2 "physical activity" ) OR ( diet* W/2 "combined with" W/2 "physical activity" ) OR ( diet* W/2 "along with" W/2 "physical activity" ) OR ( "diet and" W/2 "physical activity" ) OR ( "diet +" W/2 "physical activity" ) OR ( "diet+" W/2 "physical activity" ) OR ( "nutrition therapy" W/2 "with" W/2 "physical activity" ) OR ( "nutrition therapy" W/2 plus W/2 "physical activity" ) OR ( "nutrition therapy" W/2 "in combination with" W/2 "physical activity" ) OR ( "nutrition therapy" W/2 "combined with" W/2 "physical activity" ) OR ( "nutrition therapy" W/2 "along with" W/2 "physical activity" ) OR ( "nutrition therapy and" W/2 "physical activity" ) OR ( "nutrition therapy +" W/2 "physical activity" ) ) ) OR ( TITLE-ABS-KEY ( ( nutrition W/2 "with" W/2 training ) OR ( nutrition W/2 plus W/2 training ) OR ( nutrition W/2 "in combination with" W/2 training ) OR ( nutrition W/2 "combined with" W/2 training ) OR ( nutrition W/2 "along with" W/2 training ) OR ( "nutrition and" W/2 training ) OR ( "nutrition+" W/2 training ) OR ( "nutrition +" W/2 training ) OR ( diet* W/2 "with" W/2 training ) OR ( diet* W/2 plus W/2 training ) OR ( diet* W/2 "in combination with" W/2 training ) OR ( diet* W/2 "combined with" W/2 training ) OR ( diet* W/2 "along with" W/2 training ) OR ( "diet and" W/2 training ) OR ( "diet +" W/2 training ) OR ( "diet+" W/2 training ) OR ( "nutrition therapy" W/2 "with" W/2 training ) OR ( "nutrition therapy" W/2 plus W/2 training ) OR ( "nutrition therapy" W/2 "in combination with" W/2 training ) OR ( "nutrition therapy" W/2 "combined with" W/2 training ) OR ( "nutrition therapy" W/2 "along with" W/2 training ) OR ( "nutrition therapy and" W/2 training ) OR ( "nutrition therapy +" W/2 training ) ) ) ) ) ) ) AND NOT ( ( ( TITLE ( "Cohort study" OR genes OR mice OR birds OR fishes OR "animal experimentation" OR "Cross-Sectional Study" OR invertebrates OR qualitative AND research OR horses OR rats OR child OR infant OR adolescent ) ) OR ( TITLE ( "case control" OR "case-control" OR observational OR "Cohort study" OR "Cross-Sectional" OR "Cross Sectional" OR prevalence* OR animal* OR cohort* OR incidence OR "animal experimentation" OR "Animal Model" OR "laboratory animals" OR "Qualitative Research" OR "Qualitative study" OR in-vitro OR "in vitro" OR in-ovo OR "in vivo" OR in-vivo ) ) OR ( TITLE-ABS-KEY ( cell OR cells OR genes OR gene OR "Genetic polymorphism" OR "Polymorphism Genetic" OR mice OR bird OR birds OR fishes OR mouse OR rats OR hamster OR hamsters OR pigs OR swine OR swines OR piglets OR piglet OR guinea AND pigs OR guinea AND pig OR rabbits OR rabbit OR hares OR hare OR cats OR cat OR dogs OR dog OR canine OR canines OR canis OR sheep OR sheeps OR monkey OR monkeys OR chimpanzee OR chimpanzees OR horse OR horses OR pongo OR chicken OR chickens OR reptile OR reptilia OR reptiles OR cow OR cows OR piglets OR boar OR boars OR chickens OR mare OR mares OR equine OR rooster OR goats OR shrimp OR sheep OR crab OR ducks OR fowl OR fowls OR concurrent OR roots OR bacteria OR camel OR parakeets OR parrot OR parrots OR donkey OR donkeys OR mule OR mules OR zebra OR zebras OR shrew OR shrews OR bison OR bisons OR buffalo OR buffaloes OR deer OR deers OR bear OR bears OR panda OR pandas OR wild AND hog OR wild AND boar OR beaver OR beavers OR jerboa OR jerboas OR capybara OR capybaras OR cistron* OR genetic* OR pig* OR fish* OR child OR children OR adolescent OR adolescents ) ) ) ) ) |
| ISI web of science | ((TS=((nutrition NEAR/2 "with" NEAR/2 exercise) or (nutrition NEAR/2 plus NEAR/2 exercise) or (nutrition NEAR/2 "in combination with" NEAR/2 exercise) or (nutrition NEAR/2 "combined with" NEAR/2 exercise) or (nutrition NEAR/2 "along with" NEAR/2 exercise) or ("nutrition and" NEAR/2 exercise) or ("nutrition+" NEAR/2 exercise) or ("nutrition +" NEAR/2 exercise) or (diet$ NEAR/2 "with" NEAR/2 exercise) or (diet$ NEAR/2 plus NEAR/2 exercise) or (diet$ NEAR/2 "in combination with" NEAR/2 exercise) or (diet$ NEAR/2 "combined with" NEAR/2 exercise) or (diet$ NEAR/2 "along with" NEAR/2 exercise) or ("diet and" NEAR/2 exercise) or ("diet +" NEAR/2 exercise) or ("diet+" NEAR/2 exercise) or ("nutrition therapy" NEAR/2 "with" NEAR/2 exercise) or ("nutrition therapy" NEAR/2 plus NEAR/2 exercise) or ("nutrition therapy" NEAR/2 "in combination with" NEAR/2 exercise) or ("nutrition therapy" NEAR/2 "combined with" NEAR/2 exercise) or ("nutrition therapy" NEAR/2 "along with" NEAR/2 exercise) or ("nutrition therapy and" NEAR/2 exercise) or ("nutrition therapy +" NEAR/2 exercise)) OR TI= ((nutrition NEAR/2 "with" NEAR/2 exercise) or (nutrition NEAR/2 plus NEAR/2 exercise) or (nutrition NEAR/2 "in combination with" NEAR/2 exercise) or (nutrition NEAR/2 "combined with" NEAR/2 exercise) or (nutrition NEAR/2 "along with" NEAR/2 exercise) or ("nutrition and" NEAR/2 exercise) or ("nutrition+" NEAR/2 exercise) or ("nutrition +" NEAR/2 exercise) or (diet$ NEAR/2 "with" NEAR/2 exercise) or (diet$ NEAR/2 plus NEAR/2 exercise) or (diet$ NEAR/2 "in combination with" NEAR/2 exercise) or (diet$ NEAR/2 "combined with" NEAR/2 exercise) or (diet$ NEAR/2 "along with" NEAR/2 exercise) or ("diet and" NEAR/2 exercise) or ("diet +" NEAR/2 exercise) or ("diet+" NEAR/2 exercise) or ("nutrition therapy" NEAR/2 "with" NEAR/2 exercise) or ("nutrition therapy" NEAR/2 plus NEAR/2 exercise) or ("nutrition therapy" NEAR/2 "in combination with" NEAR/2 exercise) or ("nutrition therapy" NEAR/2 "combined with" NEAR/2 exercise) or ("nutrition therapy" NEAR/2 "along with" NEAR/2 exercise) or ("nutrition therapy and" NEAR/2 exercise) or ("nutrition therapy +" NEAR/2 exercise)) OR TS= ((nutrition NEAR/2 "with" NEAR/2 "physical activity") or (nutrition NEAR/2 plus NEAR/2 "physical activity") or (nutrition NEAR/2 "in combination with" NEAR/2 "physical activity") or (nutrition NEAR/2 "combined with" NEAR/2 "physical activity") or (nutrition NEAR/2 "along with" NEAR/2 "physical activity") or ("nutrition and" NEAR/2 "physical activity") or ("nutrition+" NEAR/2 "physical activity") or ("nutrition +" NEAR/2 "physical activity") or (diet$ NEAR/2 "with" NEAR/2 "physical activity") or (diet$ NEAR/2 plus NEAR/2 "physical activity") or (diet$ NEAR/2 "in combination with" NEAR/2 "physical activity") or (diet$ NEAR/2 "combined with" NEAR/2 "physical activity") or (diet$ NEAR/2 "along with" NEAR/2 "physical activity") or ("diet and" NEAR/2 "physical activity") or ("diet +" NEAR/2 "physical activity") or ("diet+" NEAR/2 "physical activity") or ("nutrition therapy" NEAR/2 "with" NEAR/2 "physical activity") or ("nutrition therapy" NEAR/2 plus NEAR/2 "physical activity") or ("nutrition therapy" NEAR/2 "in combination with" NEAR/2 "physical activity") or ("nutrition therapy" NEAR/2 "combined with" NEAR/2 "physical activity") or ("nutrition therapy" NEAR/2 "along with" NEAR/2 "physical activity") or ("nutrition therapy and" NEAR/2 "physical activity") or ("nutrition therapy +" NEAR/2 "physical activity")) OR TI= ((nutrition NEAR/2 "with" NEAR/2 "physical activity") or (nutrition NEAR/2 plus NEAR/2 "physical activity") or (nutrition NEAR/2 "in combination with" NEAR/2 "physical activity") or (nutrition NEAR/2 "combined with" NEAR/2 "physical activity") or (nutrition NEAR/2 "along with" NEAR/2 "physical activity") or ("nutrition and" NEAR/2 "physical activity") or ("nutrition+" NEAR/2 "physical activity") or ("nutrition +" NEAR/2 "physical activity") or (diet$ NEAR/2 "with" NEAR/2 "physical activity") or (diet$ NEAR/2 plus NEAR/2 "physical activity") or (diet$ NEAR/2 "in combination with" NEAR/2 "physical activity") or (diet$ NEAR/2 "combined with" NEAR/2 "physical activity") or (diet$ NEAR/2 "along with" NEAR/2 "physical activity") or ("diet and" NEAR/2 "physical activity") or ("diet +" NEAR/2 "physical activity") or ("diet+" NEAR/2 "physical activity") or ("nutrition therapy" NEAR/2 "with" NEAR/2 "physical activity") or ("nutrition therapy" NEAR/2 plus NEAR/2 "physical activity") or ("nutrition therapy" NEAR/2 "in combination with" NEAR/2 "physical activity") or ("nutrition therapy" NEAR/2 "combined with" NEAR/2 "physical activity") or ("nutrition therapy" NEAR/2 "along with" NEAR/2 "physical activity") or ("nutrition therapy and" NEAR/2 "physical activity") or ("nutrition therapy +" NEAR/2 "physical activity")) OR TS= ((nutrition NEAR/2 "with" NEAR/2 training) or (nutrition NEAR/2 plus NEAR/2 training) or (nutrition NEAR/2 "in combination with" NEAR/2 training) or (nutrition NEAR/2 "combined with" NEAR/2 training) or (nutrition NEAR/2 "along with" NEAR/2 training) or ("nutrition and" NEAR/2 training) or ("nutrition+" NEAR/2 training) or ("nutrition +" NEAR/2 training) or (diet$ NEAR/2 "with" NEAR/2 training) or (diet$ NEAR/2 plus NEAR/2 training) or (diet$ NEAR/2 "in combination with" NEAR/2 training) or (diet$ NEAR/2 "combined with" NEAR/2 training) or (diet$ NEAR/2 "along with" NEAR/2 training) or ("diet and" NEAR/2 training) or ("diet +" NEAR/2 training) or ("diet+" NEAR/2 training) or ("nutrition therapy" NEAR/2 "with" NEAR/2 training) or ("nutrition therapy" NEAR/2 plus NEAR/2 training) or ("nutrition therapy" NEAR/2 "in combination with" NEAR/2 training) or ("nutrition therapy" NEAR/2 "combined with" NEAR/2 training) or ("nutrition therapy" NEAR/2 "along with" NEAR/2 training) or ("nutrition therapy and" NEAR/2 training) or ("nutrition therapy +" NEAR/2 training)) OR TI= ((nutrition NEAR/2 "with" NEAR/2 training) or (nutrition NEAR/2 plus NEAR/2 training) or (nutrition NEAR/2 "in combination with" NEAR/2 training) or (nutrition NEAR/2 "combined with" NEAR/2 training) or (nutrition NEAR/2 "along with" NEAR/2 training) or ("nutrition and" NEAR/2 training) or ("nutrition+" NEAR/2 training) or ("nutrition +" NEAR/2 training) or (diet$ NEAR/2 "with" NEAR/2 training) or (diet$ NEAR/2 plus NEAR/2 training) or (diet$ NEAR/2 "in combination with" NEAR/2 training) or (diet$ NEAR/2 "combined with" NEAR/2 training) or (diet$ NEAR/2 "along with" NEAR/2 training) or ("diet and" NEAR/2 training) or ("diet +" NEAR/2 training) or ("diet+" NEAR/2 training) or ("nutrition therapy" NEAR/2 "with" NEAR/2 training) or ("nutrition therapy" NEAR/2 plus NEAR/2 training) or ("nutrition therapy" NEAR/2 "in combination with" NEAR/2 training) or ("nutrition therapy" NEAR/2 "combined with" NEAR/2 training) or ("nutrition therapy" NEAR/2 "along with" NEAR/2 training) or ("nutrition therapy and" NEAR/2 training) or ("nutrition therapy +" NEAR/2 training))) AND (TI= ("Clinical trial" or "clinical trial, phase i" or "clinical trial, phase ii" or clinical trial, phase iii or clinical trial, phase iv or controlled clinical trial or "multicenter study" or “clinical trial” or "randomized controlled trial" or "random allocation" or "clinical trials as topic" or randomized or random* or intervention or trial or placebo* or randomised or controlled or "4 arm" or "four arm" or single or doubl* or tripl* or treb* or blind* or mask*) OR TI= ((randomi?ed NEAR/7 trial*) or (controlled NEAR/3 trial*) or (clinical NEAR/2 trial*)))) NOT TI= ("case control" or case-control or observational or "Cohort study" or "Cross-Sectional" or "Cross Sectional" or prevalence* or animal* or cohort* or incidence or "Cross Sectional" or "animal experimentation" or "Animal Model" or "laboratory animal" or "Qualitative Research" or "Qualitative study" or in-vitro or "in vitro" or in-ovo or "in vivo" or in-vivo or cell or cells or genes or gene or "Genetic polymorphism" or "Polymorphism, Genetic" or Mice or bird or birds or fishes or mouse or rats or hamster or hamsters or pigs or swine or swines or piglets or piglet or guinea pigs or guinea pig or rabbits or rabbit or hares or hare or cats or cat or dogs or dog or canine or canines or canis or sheep or sheeps or monkey or monkeys or chimpanzee or chimpanzees or horse or horses or pongo or chicken or chickens or reptile or reptilia or reptiles or cow or cows or piglets or boar or boars or chickens or mare or mares or equine or Rooster or goats or shrimp or sheep or crab or ducks or fowl or fowls or Concurrent or roots or bacteria or camel or parakeets or parrot or parrots or donkey or donkeys or mule or mules or zebra or zebras or shrew or shrews or bison or bisons or buffalo or buffaloes or deer or deers or bear or bears or panda or pandas or wild hog or wild boar or beaver or beavers or jerboa or jerboas or capybara or capybaras or cistron* or genetic* or pig* or Fish* or child or children or adolescent or adolescents) |
